# Mining and Validating Social Media Data for COVID-19–Related Human Behaviors Between January and July 2020: Infodemiology Study

**DOI:** 10.2196/27059

**Published:** 2021-05-25

**Authors:** Ashlynn R Daughton, Courtney D Shelley, Martha Barnard, Dax Gerts, Chrysm Watson Ross, Isabel Crooker, Gopal Nadiga, Nilesh Mukundan, Nidia Yadira Vaquera Chavez, Nidhi Parikh, Travis Pitts, Geoffrey Fairchild

**Affiliations:** 1 Analytics, Intelligence, and Technology Los Alamos National Laboratory Los Alamos, NM United States; 2 Computer Science University of New Mexico Albuquerque, NM United States

**Keywords:** Twitter, social media, human behavior, infectious disease, COVID-19, coronavirus, infodemiology, infoveillance, social distancing, shelter-in-place, mobility, COVID-19 intervention

## Abstract

**Background:**

Health authorities can minimize the impact of an emergent infectious disease outbreak through effective and timely risk communication, which can build trust and adherence to subsequent behavioral messaging. Monitoring the psychological impacts of an outbreak, as well as public adherence to such messaging, is also important for minimizing long-term effects of an outbreak.

**Objective:**

We used social media data from Twitter to identify human behaviors relevant to COVID-19 transmission, as well as the perceived impacts of COVID-19 on individuals, as a first step toward real-time monitoring of public perceptions to inform public health communications.

**Methods:**

We developed a coding schema for 6 categories and 11 subcategories, which included both a wide number of behaviors as well codes focused on the impacts of the pandemic (eg, economic and mental health impacts). We used this to develop training data and develop supervised learning classifiers for classes with sufficient labels. Classifiers that performed adequately were applied to our remaining corpus, and temporal and geospatial trends were assessed. We compared the classified patterns to ground truth mobility data and actual COVID-19 confirmed cases to assess the signal achieved here.

**Results:**

We applied our labeling schema to approximately 7200 tweets. The worst-performing classifiers had F1 scores of only 0.18 to 0.28 when trying to identify tweets about monitoring symptoms and testing. Classifiers about social distancing, however, were much stronger, with F1 scores of 0.64 to 0.66. We applied the social distancing classifiers to over 228 million tweets. We showed temporal patterns consistent with real-world events, and we showed correlations of up to –0.5 between social distancing signals on Twitter and ground truth mobility throughout the United States.

**Conclusions:**

Behaviors discussed on Twitter are exceptionally varied. Twitter can provide useful information for parameterizing models that incorporate human behavior, as well as for informing public health communication strategies by describing awareness of and compliance with suggested behaviors.

## Introduction

Health authorities can minimize the impact of an emergent infectious disease through effective and timely risk communication, vaccines and antiviral therapies, and the promotion of health behaviors, such as social distancing and personal hygiene practices [1-4]. Of these, official communication is the earliest available strategy, and its effectiveness will build trust and adherence to the remaining measures [1]. During the H1N1 influenza pandemic in 2009, most countries focused on the promotion of health behaviors [2] such as mask-wearing, avoidance of crowds, and increased disinfection after observing that such protocols contributed substantially to reduced transmission and ultimate control of disease during the SARS outbreak in 2003 [5]. Health authorities have paid less attention to the psychological factors associated with a pandemic [3,4], though such factors play a vital role in subsequent adherence to health behaviors and vaccine uptake [1]. During the emergence of the Zika virus in 2016, public health guidelines focused on preventing sexual transmission by using condoms, avoiding travel to locations with active Zika transmission, and mosquito control [6], with varying levels of compliance [7,8].

Research into the use of social media and internet data for health surveillance is a growing field. Individuals discuss a wide variety of health concerns and health behaviors online, from symptom searching [9] and personal experiences with infectious diseases [2] to dieting [10] and electronic cigarette use [11]. These data have been used to identify prominent points of discussion in relation to health topics [12-14], which can point toward more effective health policies and interventions. In addition, social media and internet data reflect temporal and spatial patterns in health behavior [9-12,15]. The association between internet data and health behavior, topics, and attitudes relevant to the public provides insight into the manner in which individuals receive health information and how that information may translate into behavioral change. Specifically for disease outbreaks, internet and social media data provide opportunities for public health officials to monitor prevalent attitudes and behaviors at a given time to target further interventions and policies.

In this work, we used social media data to better understand human behaviors relevant to COVID-19 transmission and the perceived impacts of COVID-19 on individuals. We developed a coding schema for 6 categories and 11 subcategories, which included both a wide number of behaviors as well as codes focused on the impacts of the pandemic (eg, economic and mental health impacts). We applied this schema to approximately 7200 tweets and developed supervised learning classifiers for classes with sufficient labels. We then applied these classifiers to an extensive Twitter data set and showed patterns in human behaviors temporally and spatially across the United States.

We specifically focused on the following research questions:

1. Research Question 1: What behaviors related to COVID-19 are discussed on social media websites, specifically Twitter? Using content analysis techniques similar to other social media studies (eg, Ramanadhan et al [16] and Carrotte et al [17]), we identified behaviors discussed on Twitter that could be relevant to disease transmission or the downstream impacts of COVID-19. At the outset, we were particularly interested in social distancing, hygiene, and personal protective equipment practices, but we were also interested in identifying the breadth of behaviors that might be discussed.
2. Research Question 2: How do patterns in behaviors change geospatially and temporally in the United States? Using labeled data from Research Question 1, we built classification models to identify behaviors in the larger Twitter corpus. We were interested in temporal and geospatial trends in these classified data with the goal of observing regional patterns and temporal changes that occurred in conjunction with real-world events. Prior work has used similar methods to observe patterns during Zika emergence in 2016 [15].
3. Research Question 3: How do these trends compare to other data streams, like mobility data sets? Prior work has shown that social media data are biased in multiple ways [18,19]. One way to validate our findings is to compare results using social media data to other data sources that have been useful to measure human behavior during the COVID-19 pandemic. In particular, several studies have shown that mobility data sets that rely on mobile devices (eg, smartphones) have been useful at accurately gauging reduced mobility [20,21].

## Methods

### Data

For this work, we used a data set of tweets provided by Chen et al [22]. Data collection started on January 28, 2020, and used Twitter’s search application programming interface (API) to get historical tweets as early as January 21, 2020. They started with 14 keywords related to the novel coronavirus, and later expanded both keywords and individual accounts tracked over time. The data relied on Twitter’s streaming API, and are thus a 1% sample of tweets that include the keywords. The original repository contained about 270 million tweets as of mid-July 2020 [22]. Of these, we were able to collect 84% (N=228,030,309).

### Schema Development

**Figure 1:**
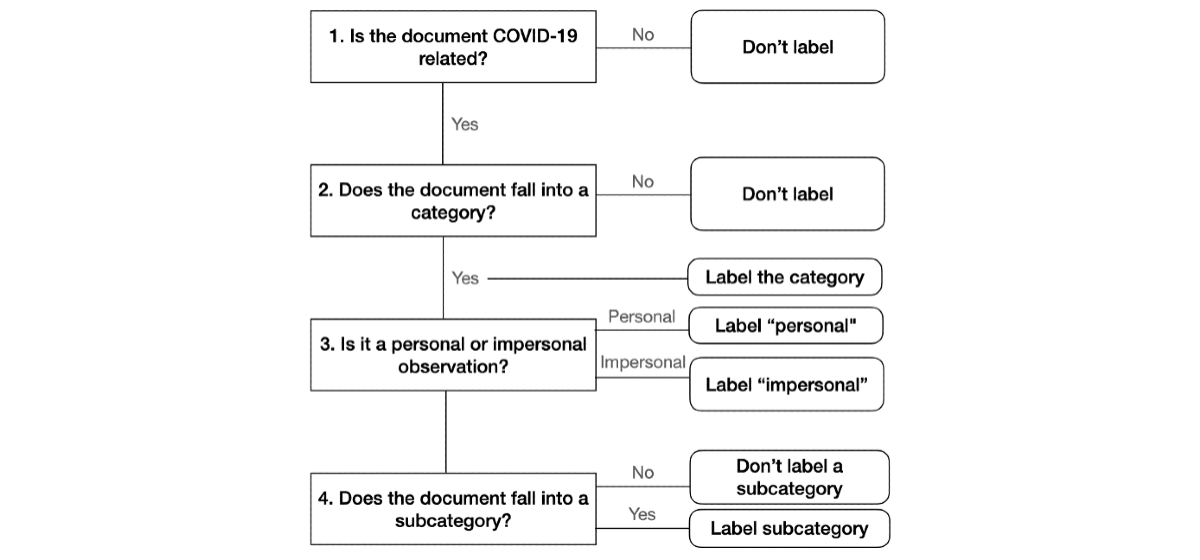
Decision tree for labeling.

The coding schema was developed by three of the authors (AD, DG, and CS) through iterative analysis of random samples of tweets from our corpus. We started initially with categories of interest (eg, social distancing and personal protective equipment) and added both categories and subcategories as they were identified in tweets, similar to prior work [16,17]. The final schema is hierarchical, where annotators can label categories and, if applicable, subcategories within the category of interest (Figure 1).

Personal and impersonal viewpoints were labeled separately from the tweet category. Here, a *personal viewpoint* is a tweet that describes a direct observation of the behavior, meaning the individual tweeting talks about their own behavior, or a person or event that the user can directly observe. For example, the tweet “I am wearing a mask when I go out” is a personal mention of personal protective equipment, specifically mask-wearing. An *impersonal viewpoint*, in contrast, includes actions like sharing articles, retweeting, or expressing an opinion without providing evidence that the user themself engages in the behavior (eg, “Ugh, I wish more people wore masks”). This definition is the same as prior work [15]. Of note, tweets were only labeled as personal or impersonal if they were already labeled with a category. Tweets that were outside the labels of interest were not labeled for viewpoint.

### Training Data

#### Training Annotators and Annotation

To create our training data set, 7278 tweets were selected at random from the English tweets we collected between January and May 2020, as labeling commenced in May. Using the above schema, we then trained three additional annotators. Annotators were trained using the following steps. First, a member of the team (AD) met with each individual prospective annotator and thoroughly described the schema. The prospective annotator and AD first labeled 16 example tweets together using tweets already labeled during schema development. The annotator then individually coded 160 additional tweets previously labeled by the authors. If agreement was sufficiently high (>0.6), the annotator was then given their own section of training data to code. Each tweet in our training data set was coded by two such annotators. All annotators met weekly to discuss questions about labels. All tweets with disagreements were resolved by a third annotator or via group discussion. The workflow to label tweets is given in Figure 1. Tweets can be labeled with more than one label, as applicable.

#### Annotator Agreement

Annotator agreement varied. Personal and impersonal labels had agreements of 0.41, 0.44, and 0.41 between the three pairs of annotators. Category-level labels had agreements of 0.77, 0.82, and 0.82, and subcategory-level labels had agreements of 0.61, 0.65, and 0.66. Distinguishing between personal and impersonal tweets was the hardest classification task because it is inherently difficult to correctly identify voice in the span of 280 characters, especially without additional context. Prior work has relied on the use of personal pronouns (eg, “I,” “we,” and “our”) to identify personal tweets [15], but it is clear that this method has a high false negative rate because of linguistic patterns like pronoun-drop (eg, the tweet “Went to the store today and nobody was wearing masks” drops the pronoun “I” and leaves it implied) [23]. Thus, despite the difficulty in labeling these tweets, we believe it is preferable to automated methods.

### Classification Algorithms

#### Tweet Preprocessing

Tweet URLs and usernames (@-mentions) were replaced with the tokens “URL” and “USERNAME,” respectively. Consecutive characters were truncated (eg, “greaaaaaat” was truncated to “great”) and punctuation was removed. Of the training data, 15% were reserved as the test set. Tweets were split using stratified sampling based on the category labels to preserve label proportions. Because of the small number of labels in several categories (Table 1), we only attempted to make classifiers for the following: personal or impersonal, social distancing (category), shelter-in-place (subcategory), monitoring (category), hygiene (category), and personal protective equipment (category).

Because personal and impersonal labels were only assigned to tweets if they fell into a category, the training data for this classifier were only those tweets with an initial label. In contrast, all other classifiers used binary classification and included all tweets that did not include the label of interest, including tweets with no labels. As such, all classification models were built using extremely disproportionate label distributions.

#### Logistic Regression

Logistic regression models were implemented in Python, version 3.7.7 (Python Software Foundation), using scikit-learn [24] and the elastic net penalty. Features included all unigrams, bigrams, and trigrams of tweet text. To optimize models, grid search was used with all possible combinations of the following parameters: the elastic net penalty varied the L1 ratio from 0 (equivalent to only “L2” penalty) to 1 (equivalent to only “L1” penalty), regularization strength varied in order of magnitude from 0.001 to 1000, and chi-square feature selection was varied from 10% to 100% of the features (ie, no feature selection), in steps of 10%, to explore the impact of feature reduction on model performance.

#### Random Forest

Random forest models were implemented using scikit-learn’s random forest classifier [24]. As in logistic regression, features included all unigrams, bigrams, and trigrams of tweet text. Again, grid search was used to optimize models. The minimum number of samples per leaf node was varied from 2 to 11 (in steps of 3), the minimum number of samples required to split an internal node ranged from 2 to 52 (in steps of 10), and the number of trees per forest was either 50 or 100. Last, we additionally varied the number of features. Because of the larger number of parameters tested here, we tried feature selections of 25%, 50%, 75%, or 100% of features (ie, no feature selection).

### Classification and Bias Adjustments

Both types of models performed poorly for classifying monitoring, personal protective equipment, and hygiene. As such, we did not use these models for downstream analysis. Rather, we focus on the personal or impersonal model, the social distancing classifier, and the shelter-in-place classifier.

Though random forest models sometimes produced slightly higher F1 scores, we used the logistic regression models for overall classification and downstream analysis because of the slightly higher precision values. Said another way, in this context, we preferred fewer false positives to slightly more false negatives because we were trying to identify a particular behavior and wanted as few erroneous predictions included in the classifier as possible.

To combat the bias inherent in our classifiers, as it is clear that misclassification will occur, we used the method suggested by Daughton and Paul [25] to create confidence intervals that account for classifier error. The basic principle is to use bootstrapping to generate many samples and to subsequently weight individual classifications by the positive predictive value or negative predictive value of the classifier. The bootstrapped samples are then used to generate a 95% confidence interval around the point estimate (see Daughton and Paul [25] for full details). This method has been successfully applied in similar work focused on identifying travel change behaviors in response to Zika [15]. For this work, we used 100 bootstrapped samples to generate daily confidence intervals.

### Geospatial Analysis and Comparison to Mobility and COVID-19 Data

We compared the results of our classifiers to mobility data from Descartes Labs—available at Descartes Labs [26] and described in Warren and Skillman [27]—to provide a ground truth measurement of social distancing, and to the number of confirmed COVID-19 cases in each state, as tracked by The New York Times [28]. The mobility data used geolocation services from mobile devices (eg, smartphones) to generate aggregate estimates about mobility within specific geographic areas. Descartes Labs provides data at *admin level 1* (state) mobility and *admin level 2* (county) mobility [26]. For this work, we only consider state mobility. Descartes Labs uses a normalized value of the median maximum distance traveled each day: the *m50 index*. Here, data are normalized using the median mobility per state between February 27 and March 7 (ie, a *pre–COVID-19* window). For this work, we looked at the percent change in mobility (m50 index – 100) [27], which can be interpreted as the percent change in mobility relative to the baseline period.

We used these data as a ground truth data set to validate social media tweets about social distancing and sheltering-in-place. For these comparisons, we restricted our data to those with geolocation services enabled (ie, those that used the tweet “place” to determine location), which we then aggregated by state. Here, data were aggregated to weekly data, and any weeks with fewer than 50 tweets were removed. States with fewer than 10 data points were excluded from visualization.

## Results

### Content Analysis and Labels

In total, 7278 tweets were read and labeled. Of these, 2202 tweets fell into the categories shown in Table 1. For each category and subcategory, the definition and an example anonymized tweet is shown. The most prevalent category by far was tweets about social distancing. Of these tweets, the vast majority were about sheltering-in-place, writ broadly, including tweets about adjusting to life at home (eg, work or school from home); tweets about entertainment, including hobbies and recipes; tweets about plans that were canceled (parties, weddings, etc); and a few tweets about a supposed “coronaboomer” phenomenon, where some suggested that the additional time spent at home would lead to an increase in babies born in 2021. In addition, we identified 53 tweets related to the mental health impacts of social distancing, including tweets about tactics to maintain positive mental health, as well as tweets describing the mental health difficulties associated with social distancing.

In other categories, we again saw a wide variety of health topics discussed. This included tweets about monitoring, of which roughly a third were about access to or experiences with COVID-19 testing; hygiene, including handwashing and cleaning protocols; and a few tweets (n=49) weighing in on COVID-19 vaccine development. Last, we also saw instances of tweets about the economic impacts of COVID-19, including on the supply chain and in terms of unemployment.

**Table 1:** Tweet content and relative proportion.

Category and subcategories		Definition	Example tweet (anonymized)	Tweets (N=7278), n (%)	
**Social distancing**					
	All subcategories	Discusses social distancing in either a positive or a negative way (eg, not physically seeing friends and family, not going to work, or discussing reasons why lockdowns are unnecessary)	“COVID-19 SUCKS! I can’t see my family and I really miss them.”	1494 (20.5)	
	Shelter-in-place	Discusses any aspects of shelter-in-place or stay-at-home policies; includes school or daycare (or homeschool), remote work, things to do to keep busy while staying home (eg, hobbies and recipes), canceled plans, delivery services (to avoid going out in public), and the supposed phenomenon that birth rates would increase after the pandemic (“coronaboomers”)	“State going into lockdown tomorrow. I can work from home but I’m also going to catch up on my backlog Steam library!”	1117 (15.3)	
	Mental health	Discussions about mental health; includes suggestions of activities to maintain mental health while sheltering-in-place and documents about the mental health difficulties associated with COVID-19 and social distancing	“I’m so stressed I’m going to cry. I don’t want to be where I am now, I just want to be alone for quarantine.”	53 (0.7)	
	Voting	Decisions around voting by mail (eg, for COVID-19–related safety reasons or the opposite opinion)	“Record high cases in the past few days. It’s been two weeks since the election.”	12 (0.2)	
	Hoarding	Storing things like food, medicines, and disaster supplies	“Got a bunch of masks and gloves in case the coronavirus becomes a big deal here.”	31 (0.4)	
	Public events	Descriptions of going to public places and choosing to not socially distance	“Airport security was super fast -- no lines at bag check.”	53 (0.7)	
**Monitoring**					
	All subcategories	Behavior monitoring for illness; includes monitoring friends or family that have the disease	“I keep coming across people with sore throats and cold symptoms today. Hope it’s not COVID!”	315 (4.3)	
	Testing	Ability or inability to get tested for COVID-19 infection; includes tweets expressing desires for improvements and increases in testing and novel testing strategies (eg, drive-through testing centers), or in combination with other tactics like contact tracing	“The complete failure in testing ramp up is really disappointing.”	116 (1.6)	
	Remedies	Unproven treatments, advice, and/or ways to “prevent” or “cure” the disease using natural methods (eg, vitamin D)	“Anti Neo Plastons is the natural cure for Coronavirus and your body makes them naturally!”	84 (1.2)	
Hygiene: all subcategories		Trying to prevent sickness by using good hygiene, including handwashing, cleaning and sanitation, and other cleanliness-related behaviors	“Just saw a kid about to use the water fountain. Their parent grabbed them and said ‘NOOOOOOOO… there could be COVID!’”	94 (1.3)	
Personal protective equipment: all subcategories		Using personal protective equipment to prevent illness; includes masks and gloves	“1.) Wear your mask 2.) Social distance 3.) Wash your hands! We can do this!”	164 (2.3)	
**Vaccine**					
	Provaccine	Tweets that are positive and supportive of vaccine efforts	“The work on the COVID vaccine is amazing. I can’t wait to get it!”	31 (0.4)	
	Antivaccine	Tweets that use vaccine-averse rhetoric to describe why a vaccine will be unsafe or ill-advised	“I hope you’re not in favor of the Gates vaccine. I’m not going to be tracked by a microchip!”	18 (0.2)	
**Economic**					
	Supply chain	Information or commentary about supply chain–related issues; includes information about “price gouging”	“Can we trust the food supply chain? Should we start growing our own fruits and vegetables?”	33 (0.5)	
	Unemployment	Includes descriptions of applying for unemployment benefits or commentary on the process; includes stimulus checks or commentary about unemployment or underemployment due to COVID-19	“I’m a driver for Uber, but I was put on medical leave after COVID-19 exposure & haven’t made any money since.”	53 (0.7)	

**Figure 2:**
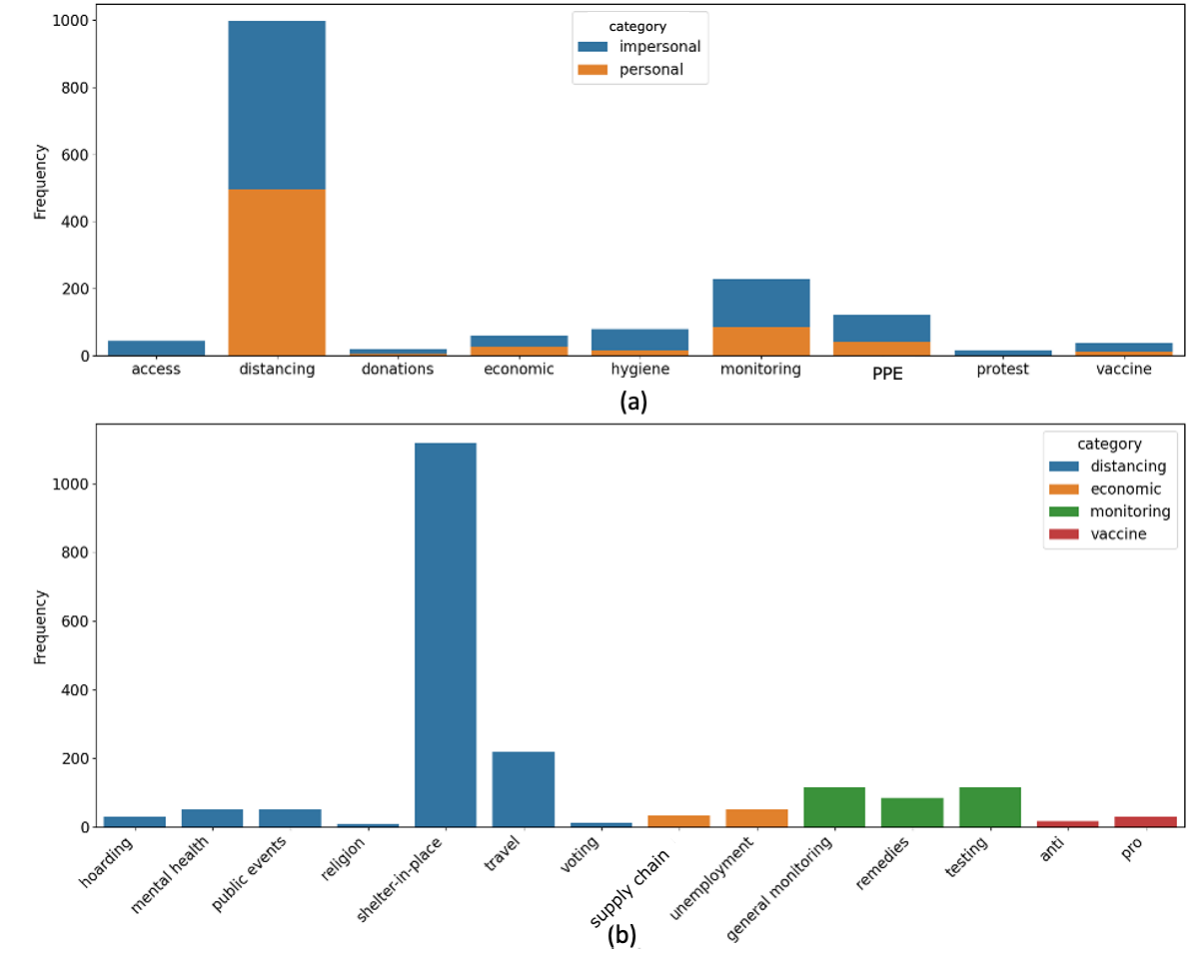
Category distribution. Tweets are broken down by frequency of personal and impersonal labels (a) and by subcategory grouped by category (b). Categories without subcategories are not shown in (b). Only categories with at least 80 labels, and subcategories with at least 50 labels, are shown. PPE: personal protective equipment.

A breakdown of categories by personal and impersonal labels is shown in Figure 2 (a), and subcategories are shown in Figure 2 (b). Overall, a small fraction of tweets were personal mentions; the majority of tweets were impersonal mentions related to each category (eg, mentions of articles or general opinions and suggestions that do not describe a personal behavior). This is consistent with prior work, which has found that personal mentions of health-related behavior on social media are rare [19].

Because there were so few tweets in most categories, it was not feasible to build robust classifiers for most categories or subcategories. For this work, we selected for classification only the personal and impersonal classification task; the categories of social distancing, monitoring, hygiene, and personal protective equipment; and the subcategory shelter-in-place. In general, we found similar performances between random forest and logistic regression (Table 2). The exception to this trend was in the personal protective category, where the logistic regression model substantially outperformed the random forest.

For subsequent analysis, we focused on categories that achieved an F1 of at least 0.6: personal or impersonal, social distancing, and the shelter-in-place classifiers. We then applied the logistic regression models to the remaining data in our corpus of over 228 million tweets through July 2020.

**Table 2:** Tweet classification results.

Classifier			Logistic regression							Random forest				
			Precision		Recall		F1 score		Precision			Recall		F1 score
Personal or impersonal			0.76		0.50		0.60		0.72			0.57		0.64
**Social distancing classifiers**														
	Social distancing (category)	0.73		0.59		0.66		0.71			0.61		0.66	
	Shelter-in-place (subcategory)	0.69		0.60		0.64		0.65			0.65		0.65	
Monitoring classifiers			0.72		0.17		0.28		0.32			0.13		0.18
Hygiene classifiers			0.50		0.29		0.36		0.33			0.21		0.26
Personal protective equipment (eg, masks and gloves) classifiers			0.59		0.52		0.55		0.40			0.24		0.30

### Temporal Patterns

**Figure 3:**
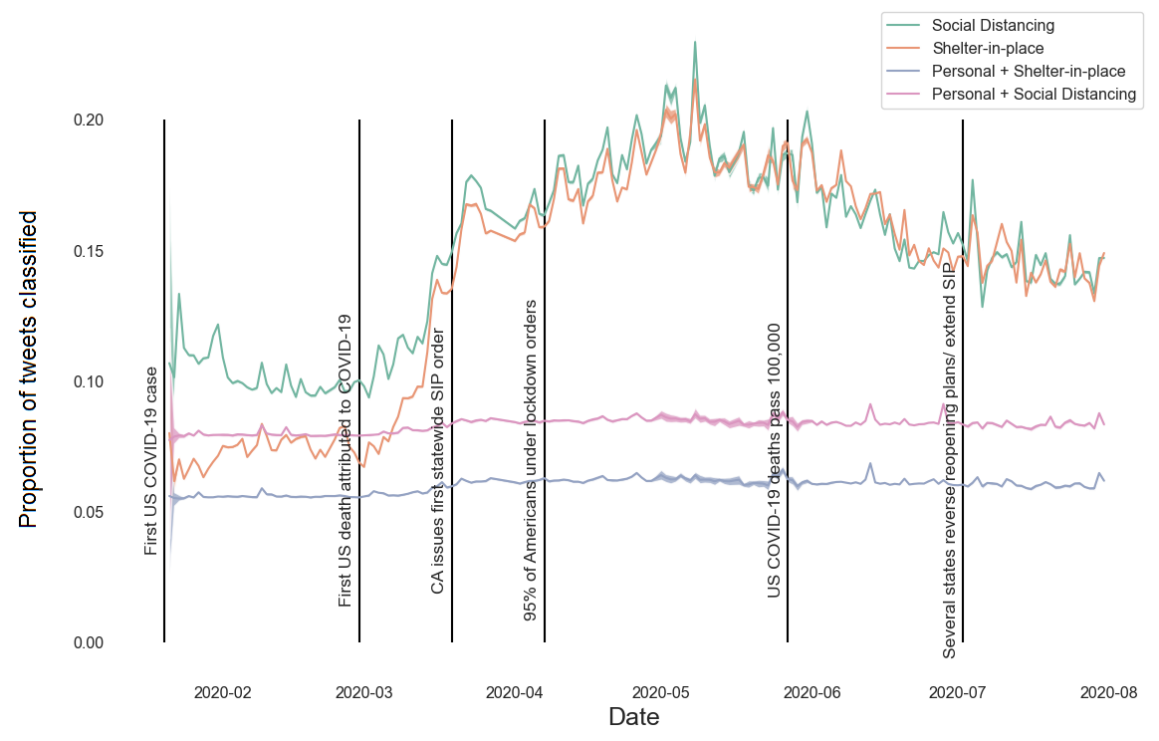
Temporal patterns in social distancing and shelter-in-place tweets. The proportion of tweets classified as general social distancing, shelter-in-place, personal shelter-in-place, and personal social distancing are shown by date. Relevant events in the outbreak are shown as vertical lines. As states increased shelter-in-place and lockdown orders, the number of tweets about social distancing and sheltering-in-place dramatically increased. Shading shows the 95% CI calculated using classifier-adjusted bootstrapped sampling while the median is a solid line. CIs are extremely small at several time points. CA: California; SIP: shelter-in-place.

Using the full classified corpus, we compared temporal patterns in social distancing tweets, shelter-in-place tweets, and the subsets of those groupings which were also classified as personal mentions, to important real-world events that occurred during the outbreak (Figure 3). Importantly, the proportion of tweets classified as social distancing and shelter-in-place tweets followed a predictable pattern with respect to real-world events occurring during the outbreak. Social distancing tweets occurred soon after the initial US COVID-19 case as people started to discuss initial reactions to the new disease. As states began to institute shelter-in-place orders—with California leading in late March 2020 [29]—the number of tweets about social distancing and sheltering-in-place doubled. Tweets in this category stayed high throughout the summer, as a large number of Americans were under shelter-in-place orders [29]. In early April 2020, estimates of the number of Americans told to stay at home were around 95%, despite widespread variation in how stay-at-home orders were implemented [30]. As expected, the number of personal tweets was a small fraction of the social distancing and shelter-in-place tweets more broadly. There was little variation in the temporal patterns of personal tweets; all signals came from the broader set of both personal and impersonal tweets.

### State Patterns: Comparisons to Mobility Data

**Figure 4:**
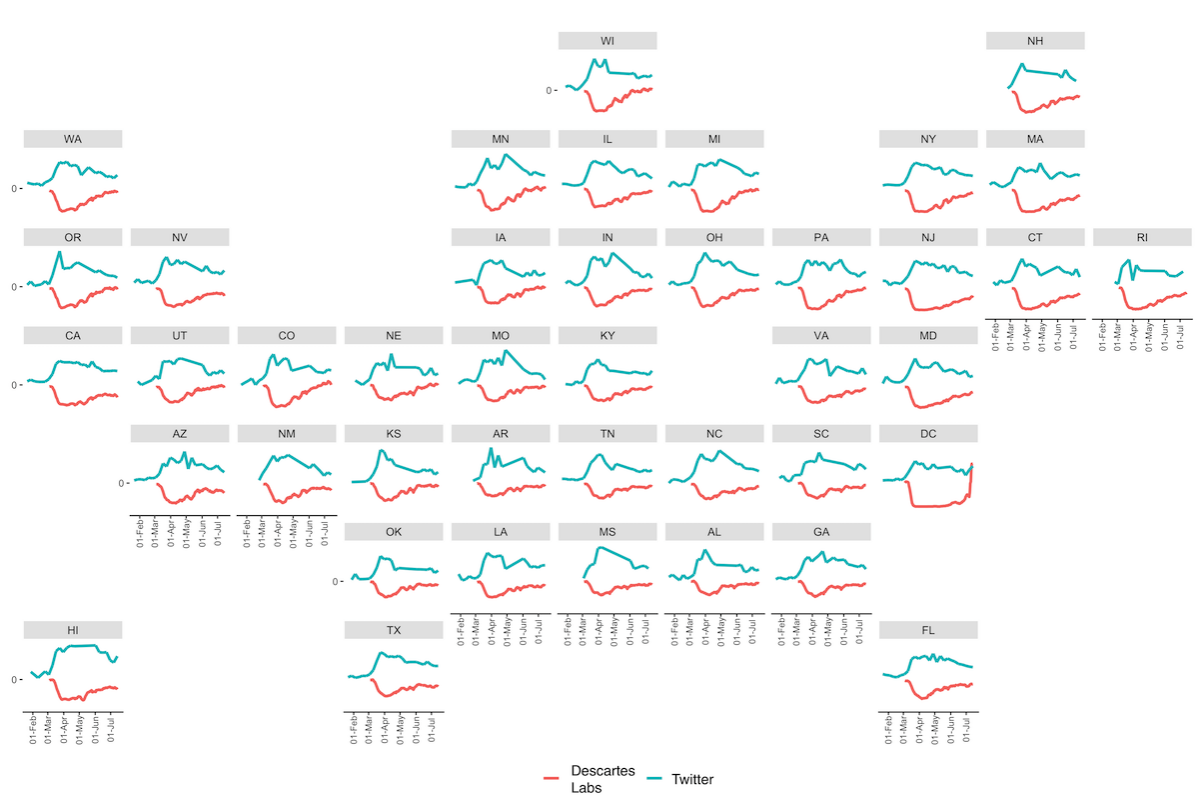
US state patterns in mobility compared to social distancing tweets from January to July 2020. Descartes Lab data showing a rolling 7-day average of percent change in mobility (divided by 5, to improve visualization) is plotted alongside the proportion of social distancing tweets per week. Both temporal and regional patterns are clear. Further, as the proportion of social distancing tweets increased, mobility measured by Descartes Labs decreased. States without sufficient Twitter data were removed from the grid. 2-letter abbreviations are used for each state.

**Figure 5:**
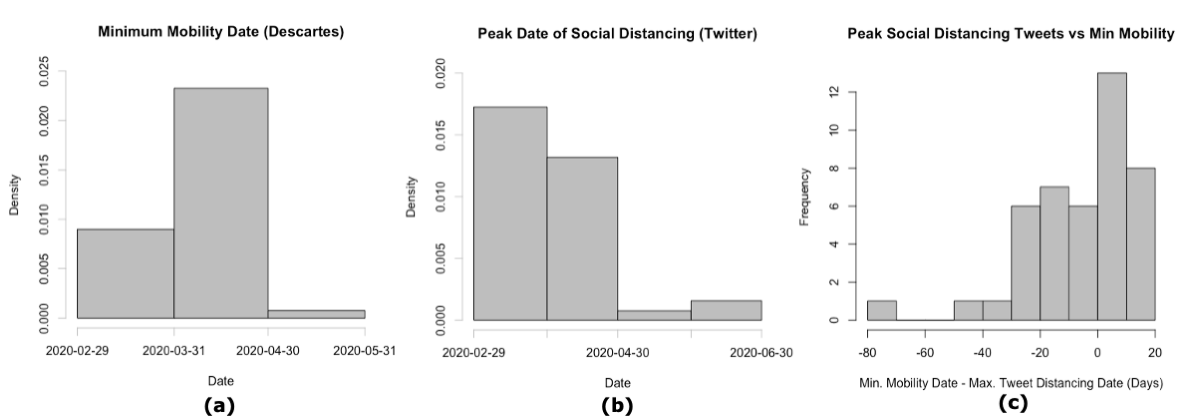
Comparison of peak social distancing tweet proportions and minimum mobility. To validate our social media findings, we compared them to mobility data provided by Descartes Labs. Dates of minimum mobility are aggregated by month (a), while dates of highest proportion of tweets about social distancing, aggregated by month, are shown in (b). The difference, in days, between the date of minimum mobility and the date of highest proportion of social distancing tweets (c) show that most states observed both peaks within 20 days of one another.

**Figure 6:**
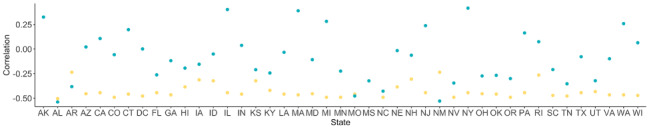
Correlation between confirmed COVID-19 cases or mobility and proportion of tweets about social distancing by US state. Most states have a moderate negative correlation between the proportion of tweets about social distancing and mobility data (yellow), indicating good agreement in the two signals. Some states have notably weaker negative correlations (eg, AR, NM, and RI), which could be the result of less Twitter data. Correlations between the number of confirmed COVID-19 cases and the proportion of tweets about social distancing are weak (blue), with a few notable exceptions (eg, AL). 2-letter abbreviations are used for each state.

To evaluate temporal patterns more closely, we considered patterns in individual states and compared them to mobility data derived from mobile phone devices (Figures 4-6) and the actual number of confirmed COVID-19 cases (Figure 6). At a high level, it is clear that there is an inverse relationship between the proportion of tweets about social distancing and the actual movement of individuals (Figure 4), indicating that social distancing conversations on Twitter may actually be reflective of real-world behavior. However, we can also see interesting regional patterns among states. For example, some of the earliest-hit states (eg, California, Washington, and New York) showed peaks in the number of tweets about social distancing in late March 2020 compared to states that saw comparatively few cases early on (eg, Florida and Georgia, which had peaks in the number of social distancing tweets in late April 2020).

Most states observed the lowest mobility in April 2020, as seen in Figure 5 (a). The day with the highest fraction of social distancing tweets was most often in March 2020, though many states observed this in April as well, as seen in Figure 5 (b). In general, most states observed these dates within ±20 days of each other, with the majority of states observing the day of minimum mobility before the day with the most tweets about social distancing, as seen in Figure 5 (c). Further, there is a strong negative correlation between the mobility data and the classified Twitter data (Figure 6). Though patterns vary by state, the average correlation is –0.42. Some states show a notably weaker signal (eg, Arkansas, New Mexico, and Rhode Island), which could be caused in part by the relative lack of data in these states. Taken together, these suggest a reasonably strong relationship between our classified Twitter data set and the ground truth mobility data. These patterns are not as clearly reflected in the relationship to confirmed COVID-19 cases. The average correlation between the proportion of tweets about social distancing and the number of confirmed COVID-19 cases is –0.08, though the strongest, which comes from Alabama, is –0.53. This suggests that, while social distancing discussions on social media are reflective of actual social distancing practices as measured by mobility data, the link to COVID-19 transmission is likely more complicated.

## Discussion

### Principal Findings

The ongoing COVID-19 outbreak clearly illustrates the need for real-time information gathering to assess evolving beliefs and behaviors that directly impact disease spread. Historically, such information would be gathered using survey methods [5,7,31], which are time-consuming, expensive, and typically lack the ability to measure temporal and spatial variation [32]. One proposed partial solution is to use internet data (eg, search query patterns and social media data), which have been shown to correspond to disease incidence in emergent infectious disease outbreaks [23,33-35], individual risk perception [1,36,37], and risk communication [38], and have been used to identify specific health behaviors [15]. During the early stages of the current COVID-19 pandemic, social media data have been used to monitor the top concerns of individuals [39,40], characterize COVID-19 awareness [41], compare social connectedness and COVID-19 hot spots [42], monitor misinformation [40,43-45], and rapidly disseminate information [46]. Last, social media has been used as an information gathering platform during periods of uncertain information. Disease emergence is a context wherein disease risks, transmission, and treatment may be largely unclear [46]. With this context in mind, we address our findings with respect to each research question below.

*What behaviors related to COVID-19 are discussed on social media websites, like Twitter?* We find that there are a wide variety of behaviors discussed on social media, including mask-wearing, hygiene (eg, handwashing), testing availability and experiences, and social distancing practices. Prior work has found evidence that mask-wearing and limited mobility were behaviors adopted to reduce disease spread during SARS [5] and that handwashing would be commonly implemented by individuals during a hypothetical pandemic influenza [47]. This prior work, however, has relied on surveys to obtain data about the behaviors that individuals implement. The use of social media to complement such work would improve both the richness and the temporal and geographic scope of the data available.

Some of the identified tweets show evidence of sensitive topics. For example, we found 53 tweets related to individuals’ mental health. Prior research has found that social media can be used to identify individuals with a variety of mental health concerns, including depression [48] and suicide [14]. As there is considerable work emerging about the substantial mental health impacts of COVID-19 (eg, increases in domestic violence [49] as well as depression and anxiety [50]), this could prove to be an important avenue for future work in this field.

Last, we found a small number of tweets (n=49) about vaccination related to COVID-19, of which roughly a third (n=18) showed a negative attitude. Importantly, this study was conducted *prior* to the authorization of any vaccines in the United States. All of the tweets considered here discuss either vaccine development or a hypothetical COVID-19 vaccine. Prior research has found similarly negative tweets during the emergence of Zika [51] and the H1N1 influenza pandemic [52]. Future work analyzing these data could provide additional insight into specific reasons that populations may be hesitant to receive the COVID-19 vaccine and could inform targeted public health messaging.

*How do patterns in behaviors change geospatially and temporally in the United States?* As expected, the patterns in tweets classified as social distancing and shelter-in-place followed extremely similar trends. These patterns corresponded to important real-world events during the outbreak, suggesting that individuals were responding to actual events and some were describing their own personal behavior. We found, however, that tweets classified as personal mentions represented a very small subset of social distancing and shelter-in-place tweets. This is not unexpected, given that prior work has shown that personal mentions of health may be extremely uncommon [20].

*How do these trends compare to other data streams, like mobility data sets, that have also shown promise in COVID-19 modeling efforts?* Despite the lack of a temporal signal in tweets labeled as personal and social distancing, there was a stronger signal when comparing classified data to Descartes Labs’ mobility data. We observed meaningful regional differences between states and saw that, in general, the peak number of tweets about social distancing happened within a few weeks of the actual measured minimum in mobility. This suggests that social media data may be used as a proxy for sensor data in appropriately data-rich contexts. Recent work using geotagged Twitter data to create social networks and analyze social distancing in the context of policy decisions found similar relationships and supports this finding [53].

### Limitations

There are a number of limitations to consider in this work. The first is that, as mentioned above, it is known that social media data are biased in a number of ways, including demographically, and that bias differs by geographic areas [18]. Further, personal mentions of health-related information on Twitter are rare [19]. These are known limitations of using internet data and could potentially explain the variations in correlation we observed between social distancing posts and actual mobility data. Importantly, however, it is difficult to assess this without extensive prospective surveys conducted at the same time as tweet collection.

Our observed wide range in correlations between the proportion of social distancing tweets and actual COVID-19 cases in individual states is an example of the ecological fallacy. State-level COVID-19 cases represent an aggregate measure of a state’s behavior, while tweets represent individual actions and observations. The available data do not allow us to probe the reasons for the variation, but a number of possible factors could be at play. Individuals’ social distancing thoughts at a specific moment in time will be influenced by contextual information about other aspects of their lives. For example, people that tweet in support of social distancing may have in-person jobs or be in high-risk groups, which could motivate them to use social media platforms to voice support for public health measures. The stronger correlation with mobility outcomes is expected by this same argument because mobility is more directly representative of individual actions.

Additionally, tweeting norms could be systematically different across the country (eg, people in different states might be more or less likely to talk about social distancing based on the policies in place and the perceived threat of COVID-19). It is also possible that there are differences in which individuals use Twitter and have geolocation services enabled in different states. In an operational context, it is hugely important to combine internet data with traditional data streams in order to provide a more complete picture of an evolving scenario. Future work should focus on targeted studies to better understand potential bias.

An additional known source of bias comes from imperfect classification. Our classifiers performed similarly to other classifiers used to identify health behaviors [15], but were clearly not perfect. To account for known classifier bias, we used an adjusted bootstrapping method from Daughton and Paul [25], which generates accurate confidence intervals despite classifier error.

We validated our work using mobility data from Descartes Labs. However, there are a number of mobility data sources available [54]. Prior work indicates that these data have similar patterns [54], but it is possible that using a different source would produce slightly different validation results.

### Conclusions

Behavior changes and policy decisions that occur early within an outbreak have the largest effects on disease dynamics [55,56]. Real-time conversations about health behaviors, in addition to other behavioral data sources such as mobility metrics or media consumption (eg, home television viewing [55]), could help improve overall knowledge and policy decisions in the early stages of an epidemic and could better capture dynamic changes caused by uncoordinated behavioral change. Using such data has the unique capability to inform public health decisions as an outbreak emerges, especially with respect to public health communication. The World Health Organization suggests a communication checklist to prepare for and minimize morbidity and mortality in the event of a pandemic [57,58]. The checklist emphasizes building public trust through early communication, even with incomplete information, and evaluating the impact of communication programs to assess whether recommendations are being followed. The use of social media streams as a simultaneous real-time measure of public sentiment toward messaging and a dynamic evaluation tool of communication effectiveness could be invaluable in minimizing effects from a future disease outbreak.

## Abbreviations

API: application programming interface

## References

[1] Taylor S (2019). The Psychology of Pandemics: Preparing for the Next Global Outbreak of Infectious Disease.

[2] Bults M, Beaujean DJ, Richardus JH, Voeten HA (2015). Perceptions and behavioral responses of the general public during the 2009 influenza A (H1N1) pandemic: A systematic review. Disaster Med Public Health Prep.

[3] Douglas PK, Douglas DB, Harrigan DC, Douglas KM (2009). Preparing for pandemic influenza and its aftermath: Mental health issues considered. Int J Emerg Ment Health.

[4] Shultz JM, Espinel Z, Flynn BW, Hoffman Y, Cohen RE (2008). Deep Prep: All-Hazards Disaster Behavioral Health Training.

[5] Lau JTF, Yang X, Pang E, Tsui HY, Wong E, Wing YK (2005). SARS-related perceptions in Hong Kong. Emerg Infect Dis.

[6] MacDonald PDM, Holden EW (2018). Zika and public health: Understanding the epidemiology and information environment. Pediatrics.

[7] Darrow W, Bhatt C, Rene C, Thomas L (2018). Zika virus awareness and prevention practices among university students in Miami: Fall 2016. Health Educ Behav.

[8] Mendoza C, Jaramillo G, Ant TH, Power GM, Jones RT, Quintero J, Alexander N, Webster J, Osorio L, Logan JG (2020). An investigation into the knowledge, perceptions and role of personal protective technologies in Zika prevention in Colombia. PLoS Negl Trop Dis.

[9] White RW, Horvitz E (2014). From health search to healthcare: Explorations of intention and utilization via query logs and user surveys. J Am Med Inform Assoc.

[10] Coogan S, Sui Z, Raubenheimer D (2018). Gluttony and guilt: Monthly trends in internet search query data are comparable with national-level energy intake and dieting behavior. Palgrave Commun.

[11] Ayers JW, Ribisl KM, Brownstein JS (2011). Tracking the rise in popularity of electronic nicotine delivery systems (electronic cigarettes) using search query surveillance. Am J Prev Med.

[12] Eichstaedt JC, Schwartz HA, Kern ML, Park G, Labarthe DR, Merchant RM, Jha S, Agrawal M, Dziurzynski LA, Sap M, Weeg C, Larson EE, Ungar LH, Seligman MEP (2015). Psychological language on Twitter predicts county-level heart disease mortality. Psychol Sci.

[13] Paul MJ, Dredze M (2011). You are what you tweet: Analyzing Twitter for public health. Proceedings of the Fifth International AAAI Conference on Weblogs and Social Media.

[14] McClellan C, Ali MM, Mutter R, Kroutil L, Landwehr J (2017). Using social media to monitor mental health discussions - Evidence from Twitter. J Am Med Inform Assoc.

[15] Daughton AR, Paul MJ (2019). Identifying protective health behaviors on Twitter: Observational study of travel advisories and Zika virus. J Med Internet Res.

[16] Ramanadhan S, Mendez SR, Rao M, Viswanath K (2013). Social media use by community-based organizations conducting health promotion: A content analysis. BMC Public Health.

[17] Carrotte ER, Prichard I, Lim MSC (2017). "Fitspiration" on social media: A content analysis of gendered images. J Med Internet Res.

[18] Mislove A, Lehmann S, Ahn Y, Onnela J, Rosenquist J (2011). Understanding the demographics of Twitter users. Proceedings of the Fifth International AAAI Conference on Weblogs and Social Media.

[19] Daughton AR, Chunara R, Paul MJ (2020). Comparison of social media, syndromic surveillance, and microbiologic acute respiratory infection data: Observational study. JMIR Public Health Surveill.

[20] Engle S, Stromme J, Zhou A (2020). Staying at home: Mobility effects of COVID-19. SSRN J.

[21] Buckee CO, Balsari S, Chan J, Crosas M, Dominici F, Gasser U, Grad YH, Grenfell B, Halloran ME, Kraemer MUG, Lipsitch M, Metcalf CJE, Meyers LA, Perkins TA, Santillana M, Scarpino SV, Viboud C, Wesolowski A, Schroeder A (2020). Aggregated mobility data could help fight COVID-19. Science.

[22] Chen E, Lerman K, Ferrara E (2020). Tracking social media discourse about the COVID-19 pandemic: Development of a public coronavirus Twitter data set. JMIR Public Health Surveill.

[23] Lamb A, Paul MJ, Dredze M (2013). Separating fact from fear: Tracking flu infections on Twitter. Proceedings of the 2013 Conference of the North American Chapter of the Association for Computational Linguistics: Human Language Technologies.

[24] Pedregosa F, Varoquaux G, Gramfort A, Michel V, Thirion B, Grisel O, Blondel M, Prettenhofer P, Weiss R, Dubourg V, Vanderplas J, Passos A, Cournapeau D, Brucher M, Perrot M, Duchesnay É (2011). Scikit-learn: Machine learning in Python. J Mach Learn Res.

[25] Daughton AR, Paul MJ (2019). Constructing accurate confidence intervals when aggregating social media data for public health monitoring. Proceedings of the International Workshop on Health Intelligence (W3PHAI 2019).

[26] Data for mobility changes in response to COVID-19. GitHub.

[27] Warren MS, Skillman SW Mobility changes in response to COVID-19. ArXiv..

[28] Coronavirus (Covid-19) data in the United States. GitHub.

[29] State “shelter-in-place” and “stay-at-home” orders. FINRA.

[30] Mervosh S, Lu D, Swales V (2020). See which states and cities have told residents to stay at home. The New York Times.

[31] Chandrasekaran N, Marotta M, Taldone S, Curry C (2017). Perceptions of community risk and travel during pregnancy in an area of Zika transmission. Cureus.

[32] Blaikie N (2009). Designing Social Research: The Logic of Anticipation. 2nd edition.

[33] Chan EH, Sahai V, Conrad C, Brownstein JS (2011). Using web search query data to monitor dengue epidemics: A new model for neglected tropical disease surveillance. PLoS Negl Trop Dis.

[34] Culotta A (2010). Towards detecting influenza epidemics by analyzing Twitter messages. Proceedings of the First Workshop on Social Media Analytics (SOMA '10).

[35] Watad A, Watad S, Mahroum N, Sharif K, Amital H, Bragazzi NL, Adawi M (2019). Forecasting the West Nile virus in the United States: An extensive novel data streams-based time series analysis and structural equation modeling of related digital searching behavior. JMIR Public Health Surveill.

[36] Hassan MS, Halbusi HA, Najem A, Razali A, Williams KA, Mustamil NM Impact of risk perception on trust in government and self-efficiency during COVID-19 pandemic: Does social media content help users adopt preventative measures?. Research Square..

[37] Oh SH, Lee SY, Han C (2020). The effects of social media use on preventive behaviors during infectious disease outbreaks: The mediating role of self-relevant emotions and public risk perception. Health Commun.

[38] Ding H, Zhang J (2010). Social media and participatory risk communication during the H1N1 flu epidemic: A comparative study of the United States and China. China Media Res.

[39] Abd-Alrazaq A, Alhuwail D, Househ M, Hamdi M, Shah Z (2020). Top concerns of tweeters during the COVID-19 pandemic: Infoveillance study. J Med Internet Res.

[40] Singh L, Bansal S, Bode L, Budakb C, Chic G, Kawintiranona K, Paddena C, Vanarsdalla R, Vragad E, Wanga Y A first look at COVID-19 information and misinformation sharing on Twitter. ArXiv..

[41] Saad M, Hassan M, Zaffar F Towards characterizing COVID-19 awareness on Twitter. ArXiv..

[42] Bailey M, Cao R, Kuchler T, Stroebel J, Wong A (2018). Social connectedness: Measurement, determinants, and effects. J Econ Perspect.

[43] Ahmed W, Vidal-Alaball J, Downing J, López Seguí F (2020). COVID-19 and the 5G conspiracy theory: Social network analysis of Twitter data. J Med Internet Res.

[44] Broniatowski DA, Paul MJ, Dredze M (2013). National and local influenza surveillance through Twitter: An analysis of the 2012-2013 influenza epidemic. PLoS One.

[45] Gerts D, Shelley CD, Parikh N, Pitts T, Watson Ross C, Fairchild G, Vaquera Chavez NY, Daughton AR (2021). "Thought I'd share first" and other conspiracy theory tweets from the COVID-19 infodemic: Exploratory study. JMIR Public Health Surveill.

[46] Chan AKM, Nickson CP, Rudolph JW, Lee A, Joynt GM (2020). Social media for rapid knowledge dissemination: Early experience from the COVID-19 pandemic. Anaesthesia.

[47] Sadique MZ, Edmunds WJ, Smith RD, Meerding WJ, de Zwart O, Brug J, Beutels P (2007). Precautionary behavior in response to perceived threat of pandemic influenza. Emerg Infect Dis.

[48] De Choudhury CM, Gamon M, Counts S, Horvitz S (2013). Predicting depression via social media. Proceedings of the Seventh International AAAI Conference on Weblogs and Social Media.

[49] Kofman YB, Garfin DR (2020). Home is not always a haven: The domestic violence crisis amid the COVID-19 pandemic. Psychol Trauma.

[50] Vindegaard N, Benros ME (2020). COVID-19 pandemic and mental health consequences: Systematic review of the current evidence. Brain Behav Immun.

[51] Ghenai A, Mejova Y (2017). Catching Zika fever: Application of crowdsourcing and machine learning for tracking health misinformation on Twitter. Proceedings of the 2017 IEEE International Conference on Healthcare Informatics (ICHI).

[52] Salathé M, Khandelwal S (2011). Assessing vaccination sentiments with online social media: Implications for infectious disease dynamics and control. PLoS Comput Biol.

[53] Porcher S, Renault T Social distancing beliefs and human mobility: Evidence from Twitter. ArXiv..

[54] Huang X, Li Z, Jiang Y, Ye X, Deng C, Zhang J, Li X (2021). The characteristics of multi-source mobility datasets and how they reveal the luxury nature of social distancing in the US during the COVID-19 pandemic. Int J Digit Earth.

[55] Schwarzinger M, Flicoteaux R, Cortarenoda S, Obadia Y, Moatti J (2010). Low acceptability of A/H1N1 pandemic vaccination in French adult population: Did public health policy fuel public dissonance?. PLoS One.

[56] Springborn M, Chowell G, MacLachlan M, Fenichel EP (2015). Accounting for behavioral responses during a flu epidemic using home television viewing. BMC Infect Dis.

[57] World Health Organization, Department of Communicable Disease Surveillance and Response (1999). WHO Guidelines for Epidemic Preparedness and Response to Measles Outbreaks.

[58] World Health Organization, Department of Communicable Disease Surveillance and Response, Global Influenza Programme (2005). WHO Checklist for Influenza Pandemic Preparedness Planning.

